# Transcriptional profiling of anthocyanin biosynthesis in variegated and non-variegated dorsal petal regions of *Impatiens uliginosa*

**DOI:** 10.1186/s12870-026-09116-2

**Published:** 2026-06-03

**Authors:** Wenqi Zhang, Huanzhi Chi, Huangwei Yu, Lingjuan Zhang, Xiaoli Zhang, Xiaoyan Luo, Haiquan Huang, Meijuan Huang

**Affiliations:** 1 College of Landscape Architecture and Horticulture Sciences, Yunnan Key Laboratory of Landscape Plant Resource Cultivation and Application, Yunnan Province Engineering Research Center for Functional Flower Resources and Industrialization, Research and Development Center of Landscape Plants and Horticulture Flowers, Southwest Forestry University, Kunming, 650224 China; 2 Yunnan Biodiversity Research Institute, Southwest Forestry University, Kunming, 650224 China; 3 Key Laboratory for Plant Biodiversity and Biogeography of East Asia, Kunming Institute of Botany, Chinese Academy of Sciences, Kunming, 650201 China

**Keywords:** *Impatiens uliginosa*, Floral variegation, Pigment content, Transcriptomics, *MYB114*

## Abstract

**Background:**

Petal variegation is an important ornamental trait in flowering plants and has major implications for both evolutionary biology, because it can promote speciation and diversity, and horticulture, because it enhances aesthetic appeal and commercial value. Here, we used *Impatiens uliginosa* as a model system and combined morphological, anatomical, physiological, biochemical, transcriptomic, and molecular approaches to investigate the mechanisms underlying petal variegation.

**Results:**

Freehand sectioning revealed that pigments were predominantly localized in the upper epidermal cells. Compared with the non-variegated dorsal petal regions, the variegated dorsal petal regions contained approximately three times as many pigment-accumulating cells; these cells were smaller, more densely arranged, and exhibited higher pigment density per unit area, collectively contributing to the darker coloration of the variegated regions. Pigment-content analysis showed that the variegated dorsal petal regions accumulated significantly higher levels of total carotenoids, anthocyanins, and flavonoids than the non-variegated dorsal petal regions. Semi-quantitative analysis further indicated that anthocyanin-related compounds, particularly delphinidin, pelargonidin, and peonidin derivatives, were enriched in the variegated dorsal petal regions.

To investigate the molecular basis of this differential pigmentation, we performed comparative RNA-seq analysis of the variegated and non-variegated dorsal petal regions and identified a suite of candidate genes potentially involved in variegation formation. Functional validation of one candidate, *IuMYB114*, was carried out using virus-induced gene silencing (VIGS). Silencing of *IuMYB114* resulted in a significant reduction in both the size and color intensity of the variegated dorsal petal regions. qRT-PCR analysis confirmed that *IuMYB114* expression in silenced plants was only 11% of that in wild-type plants. Moreover, the expression of several key anthocyanin-biosynthetic genes, including *IuANS*, *IuUFGT*, and *IuF3′5'H*, was significantly downregulated in the silenced lines.

**Conclusions:**

Taken together, these results suggest that *IuMYB114* is likely associated with the positive regulation of anthocyanin biosynthesis and may play an important role in the formation of variegated and non-variegated dorsal petal regions in *I. uliginosa*, potentially through the regulation of anthocyanin biosynthetic genes. The spatially restricted expression of *IuMYB114* may therefore contribute to differential anthocyanin deposition and, consequently, to petal variegation.

**Supplementary Information:**

The online version contains supplementary material available at 10.1186/s12870-026-09116-2.

## Introduction

Petal variegation, one of the most distinctive floral color patterns, is a highly prized ornamental trait in garden plants [1] and also plays important biological roles in pollination [2, 3]. Floral coloration is influenced by multiple factors. For instance, higher cytoplasmic pH values are associated with deeper coloration in chrysanthemum [4], and the spatial distribution of pigments directly affects the expression of variegation patterns [5]. Therefore, understanding petal epidermal cell morphology is fundamental to elucidating the mechanisms underlying variegation formation. Accumulating evidence indicates a close relationship between epidermal cell morphology and petal coloration in angiosperms. In Rosa hybrid cultivars, for example, pigments are predominantly concentrated in the upper epidermis, where the cells are conical, whereas the lower epidermal cells are flattened. This structural arrangement results in more intense coloration and lower brightness in the upper epidermis than in the lower epidermis [6]. Similarly, in the colored petals of Zantedeschia, flattened epidermal cells have been shown to influence petal coloration by altering light reflection [7].

Floral color diversity is primarily determined by four major classes of pigments: chlorophylls [8, 9], carotenoids [10, 11], flavonoids [12], and betalains [13]. Among these, anthocyanins, the major pigmented subclass of flavonoids, have one of the most thoroughly characterized biosynthetic pathways in plants [14]. To date, the genes governing anthocyanin biosynthesis have been well elucidated and can be broadly classified into two categories: structural genes and transcription factors. Structural genes encode enzymes essential for pigment biosynthesis, including chalcone synthase, chalcone isomerase, and flavanone 3-hydroxylase, among others [15]. In parallel, anthocyanin biosynthesis is tightly regulated by transcription factors that interact with cis-regulatory elements in the promoters of structural genes to modulate their expression. These regulators include members of the MYB family [16–18], bHLH family [19–21], and WD40 repeat protein family [22–24]. By fine-tuning the expression of anthocyanin biosynthetic genes in specific petal regions, these transcription factors orchestrate the spatial deposition of anthocyanins, ultimately giving rise to variegation patterns.

Among the transcription factors regulating anthocyanin biosynthesis, MYB transcription factors are the most extensively studied and play a central role in controlling pigmentation pattern formation [25, 26]. The C1 gene isolated from maize (*Zea mays*) was the first MYB gene identified in plants [27]. In apple, *MdMYB1* and *MdMYBA* markedly enhance anthocyanin accumulation in the fruit skin by activating *UFGT* expression [28]. Yamagishi investigated the formation of splash spots on the tepals of Asiatic hybrid lilies (*Lilium* spp.) and showed that *LhMYB12-Lat* and *MYB19Long* determine spot formation on lily tepals [29]. Studies of anthocyanin biosynthesis in *Arabidopsis* have demonstrated that overexpression of *MYB113* and *MYB114* significantly increases anthocyanin accumulation [30]. Collectively, these findings indicate that variegation patterns are orchestrated by MYB transcription factors through tissue-specific positive or negative regulation of one or more structural genes, thereby modulating floral coloration and patterning.

*Impatiens uliginosa* is an annual or perennial herbaceous species in the family Balsaminaceae and the genus *Impatiens*, and it possesses abundant germplasm resources [31]. It exhibits distinctive floral morphology, including lip petals, wing petals, a dorsal petal, and a floral spur, all of which contribute to its high ornamental value. Its flower colors range from pink and white to deep red [32], and petal variegation represents one of its most important ornamental traits. To date, research on *I. uliginosa* has primarily focused on flower color [3], flower development [33], spur development [34, 35], and lignin biosynthesis [36]. However, the mechanisms underlying petal variegation in this species have received limited attention.

Preliminary observations by our research group identified a variegated phenotype in *I. uliginosa*, characterized by a distinct, regularly shaped pigmented patch on the dorsal petal that exhibits deeper coloration than the surrounding tissue. To date, studies on the mechanisms underlying petal variegation in *Impatiens* species remain scarce, underscoring the significance of investigating variegation formation in *I. uliginosa*. In this study, we employed an integrated approach combining colorimetric measurement, cell morphology observation, pigment content analysis, transcriptomic profiling, and virus-induced gene silencing (VIGS) to elucidate the mechanisms underlying petal variegation. Our findings not only have important implications for novel cultivar breeding and germplasm innovation in *Impatiens*, but also provide technical support and a theoretical foundation for understanding the molecular regulatory mechanisms of petal variegation formation in herbaceous plants.

## Materials and methods

### Plant materials

**Fig. 1:**
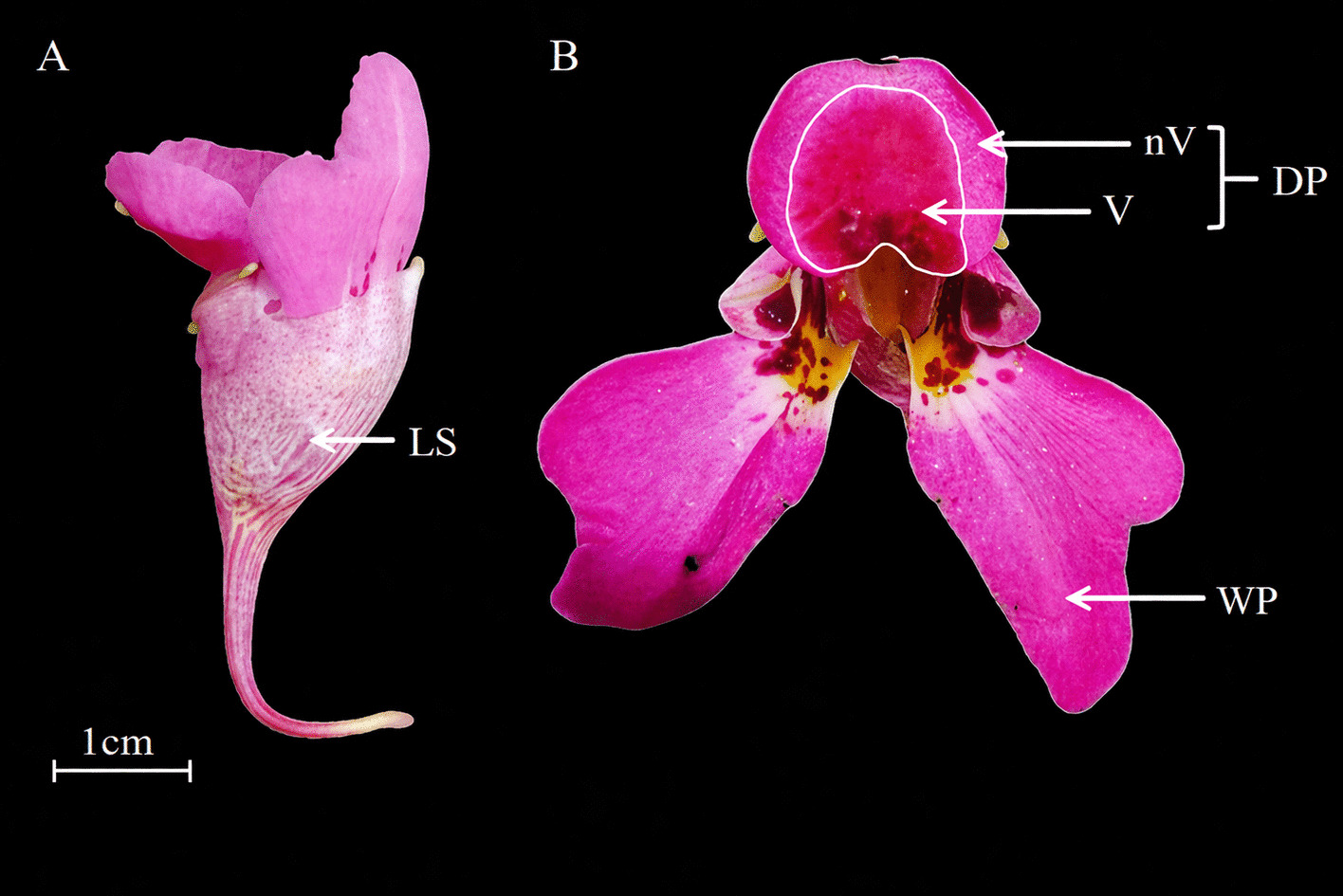
Floral morphology and spatial patterning of anthocyanin-based variegation in *I. uliginosa*. **A** Lateral view of the flower showing the characteristic saccate lower sepal (LS) and zygomorphic floral architecture. **B** Frontal view of the corolla displaying the dorsal petal (DP) with distinct variegation zones. The central variegated area (V) exhibits deep purple-red pigmentation due to high anthocyanin accumulation, whereas the peripheral non-variegated area (nV) appears pale pink. The white contour line indicates the sampling boundary between V and nV for subsequent metabolite quantification and cellular analysis. WP, wing petal; LS, lower sepal; DP, dorsal petal; V, variegated area; nV, non-variegated area. Scale bar = 1 cm

The plant material used in this study, variegated *I. uliginosa* (Fig. 1), was cultivated in the arboretum of Southwest Forestry University. Seeds of the variegated plants were originally collected from Qingshui Sea in Xundian County, Kunming, Yunnan Province, China. This lake is a plateau fault lake located at approximately 25°29′ N, 102°16′ E, at an elevation of about 2,180 m above sea level. All sampling procedures were conducted in strict accordance with the Kunming Qingshuihai Protection Regulations and other applicable water-source protection laws and regulations. As shown in Fig. 1, nV and V indicate the non-variegated and variegated areas of the dorsal petal, respectively; WP denotes the wing petal; and LS indicates the lower sepal.

### Measurement of chromaticity values

At full anthesis, the chromaticity parameters of the variegated (V) and non-variegated (nV) areas of the dorsal petals of variegated *I. uliginosa* flowers were measured using a portable spectrophotometer. Fresh petals were collected from 30 plants with uniform growth and no visible symptoms of pest infestation or disease. All measurements were conducted between 09:00 and 10:00 under standardized conditions. Colorimetric analysis was performed in the CIELAB color space using a portable spectrophotometer equipped with a standard CIE D65 illuminant and a CIE 10° standard observer. Each sample was measured in triplicate. In this color system, the lightness coordinate (L*) ranges from 0 (black) to 100 (white); the red-green coordinate (a*) indicates red (positive values) or green (negative values); and the yellow-blue coordinate (b*) indicates yellow (positive values) or blue (negative values). Chroma (C*), which reflects color saturation, was calculated as C* = (a*^2^ + b*^2^)^1/2.

### Observation of cell morphology

#### Preparation and observation of free-hand sections

Freehand sections were prepared from the dorsal petals of variegated *I. uliginosa* to examine epidermal cell morphology and pigment distribution, and representative images were captured. For each tissue region (variegated and non-variegated), three fields of view were selected. Within each field, three areas of equal size were randomly chosen for cell counting. All images were uniformly magnified to 200 ×, with a field area of 200 × 200 μm. Only pigment-containing cells were counted. The variegated and non-variegated areas were designated V and nV, respectively.

#### Preparation and observation of paraffin sections for petal epidermal cell morphology

Dorsal petals of variegated *I. uliginosa* were fixed in FAA solution (90 mL of 50% ethanol, 5 mL of glacial acetic acid, and 5 mL of 37% formaldehyde). Conventional paraffin sectioning was performed according to the method described by Li et al. [37]. Sections were cut to a thickness of 10 μm using a rotary microtome (LEICA RM205) and stained with 1% (w/v) safranin solution. Observations and image acquisition were carried out using a light microscope (LEICA DMi 1).

#### Determination of pigment content

Total anthocyanin, total flavonoid, and total carotenoid contents in the variegated and non-variegated areas of *I. uliginosa* dorsal petals were quantified according to the method described by Wan et al. [38]. Subsequently, semi-quantitative analysis of pigment aglycones in both areas was performed using high-performance liquid chromatography coupled with a diode-array detector (HPLC–DAD).

### Transcriptomic analysis

#### RNA sequencing and de novo assembly

Following the method described by Li et al. [34], tissue samples were collected from the variegated (V) and non-variegated (nV) areas of *I. uliginosa* dorsal petals at full anthesis. Petals were detached from the plants and immediately frozen in liquid nitrogen. Each sample consisted of tissue pooled from more than three biological replicates. Total RNA was extracted from plant tissue using Plant RNA Purification Reagent (Invitrogen, Carlsbad, CA, USA), and genomic DNA was removed using DNase I (Takara). RNA-seq transcriptome libraries were constructed using the TruSeq RNA Sample Preparation Kit (Illumina, San Diego, CA, USA). After quantification with a TBS380 fluorometer (Picogreen), the libraries were sequenced on an Illumina HiSeq X Ten/NovaSeq 6000 platform (Illumina, San Diego, CA, USA) to generate 2 × 150 bp paired-end reads.

Raw paired-end sequencing reads were processed for adapter trimming and quality control using SeqPrep (https://github.com/jstjohn/SeqPrep) and Sickle (https://github.com/najoshi/sickle) with default parameters. De novo assembly of the clean reads was performed using Trinity (http://trinityrnaseq.sourceforge.net/) [39]. The assembled transcripts were clustered based on sequence homology, and the longest transcript within each cluster was designated as a unigene. Assembly quality was assessed and optimized using TransRate (http://hibberdlab.com/transrate/), followed by the removal of redundant and highly similar sequences using CD-HIT (http://weizhongli-lab.org/cd-hit/). The completeness of the transcriptome assembly was evaluated using Benchmarking Universal Single-Copy Orthologs (BUSCO, http://busco.ezlab.org).

#### Functional annotation

For functional annotation and categorization, unigene sequences were aligned against multiple public databases using BLASTX (Basic Local Alignment Search Tool) with an E-value threshold of ≤ 1e-5. Annotation information was obtained by comparing the sequences against six major databases: Gene Ontology (GO), Kyoto Encyclopedia of Genes and Genomes (KEGG), Swiss-Prot, eggNOG, Pfam, and the Non-Redundant (NR) database.

#### Identification and clustering analysis of transcription factors

Transcription factor prediction and family classification were performed by aligning transcript sequences against the Plant Transcription Factor Database (PlantTFDB; http://planttfdb.cbi.pku.edu.cn/) using HMMER to identify homologous transcription factors. Hierarchical clustering analysis was subsequently performed on the differentially expressed transcription factors.

#### Screening and enrichment analysis of differentially expressed genes

Gene and transcript expression levels were quantified using TPM (transcripts per million) and FPKM (fragments per kilobase of transcript per million mapped reads), with abundance estimation performed using RSEM (http://deweylab.biostat.wisc.edu/rsem/) [40]. Differential expression analysis was conducted using DESeq2 [41], DEGseq [42], or edgeR [43]. Genes meeting the criteria of Q-value ≤ 0.05 and |log2FC|> 1 (for DESeq2 or edgeR) or Q-value ≤ 0.001 (for DEGseq) were considered significantly differentially expressed.

Functional enrichment analysis of differentially expressed genes was performed for Gene Ontology (GO) terms and Kyoto Encyclopedia of Genes and Genomes (KEGG) pathways. GO enrichment analysis was conducted using GOATOOLS (https://github.com/tanghaibao/GOatools), and KEGG pathway analysis was performed using KOBAS (http://kobas.cbi.pku.edu.cn/home.do) [44]. Statistical significance was assessed using Fisher's exact test, and P values were corrected using the Bonferroni and Benjamini–Hochberg false discovery rate (FDR) methods. Corrected P values < 0.05 were considered statistically significant.

#### Functional validation of *IuMYB114* in regulating variegation formation in *I. uliginosa*

The VIGS silencing vector was constructed according to the method described by Chen et al. [12]. Specific primers were designed based on sequence information obtained from the NCBI database. The full-length coding sequence (CDS) of *IuMYB114* was cloned into the pTRV2 vector using XbaI and BamHI restriction sites. After double digestion, ligation, and transformation into Escherichia coli DH5α, the recombinant plasmid was confirmed by colony PCR and sequencing. The verified construct was then introduced into Agrobacterium tumefaciens strain GV3101 to generate the pTRV2-*IuMYB114* recombinant vector. Plants infiltrated with the empty pTRV2 vector served as the positive control (PC), whereas those transformed with pTRV2-*IuMYB114* were designated SP1.

Agrobacterium-mediated infiltration was carried out according to the method described by Gao et al. [45], with minor modifications. Positive clones harboring pTRV1, pTRV2, and pTRV2-*IuMYB114* were cultured individually at 28 °C with shaking at 200 rpm until an OD600 of 0.5–0.8 was reached. Bacterial cells were pelleted by centrifugation at 4,000 rpm for 10 min and resuspended in infiltration buffer (10 mM MES, 10 mM MgCl2, 200 μM acetosyringone, pH 5.6) to a final OD600 of 1.5. The three suspensions were mixed at a 1:1:1 ratio and incubated in the dark at room temperature for 3–4 h. Cuttings of variegated *I. uliginosa* at the bud stage were selected for infiltration. The bacterial mixture was injected into the stem and was also used to soak the flower buds via stem injection. A total of 30 plants were infiltrated. After infiltration, the plants were kept in the dark at 8 °C for the first two days, moved to 23 °C on day 3, and then transferred to normal light conditions. Phenotypic changes were monitored and documented every three days over a 15-day period.

## Results

### Analysis of color difference values between variegated and non-variegated areas of *I. uliginosa*

Color parameters of the variegated and non-variegated areas in *I. uliginosa* were measured and statistically analyzed. As shown in Table 1, significant differences were detected in the lightness parameter (L*) between the two tissue types. The L* value of the variegated areas was 41.13 ± 2.70, significantly lower than that of the non-variegated areas (50.73 ± 0.49, *P* < 0.01), indicating darker coloration in the variegated regions. The yellow-blue parameter (b*) also differed markedly between the two areas, with a value of −8.42 ± 0.70 in the variegated regions and −16.08 ± 0.26 in the non-variegated regions (*P* < 0.001). Moreover, as illustrated in Figure S5, the variegated and non-variegated areas formed two distinct clusters based on L* values: those of the variegated areas were concentrated between 42 and 44, whereas those of the non-variegated areas ranged from 48 to 50, with no overlap between groups, further confirming the visual distinction. The red-green parameter (a*) was 33.75 ± 3.59 in the variegated areas and 26.07 ± 0.56 in the non-variegated areas. Collectively, these parameter differences indicate that the variegated areas exhibit a darker, more purplish-red hue, consistent with the observed phenotype.

**Table 1:** Analysis of chromaticity values in variegated and non-variegated areas of *I. uliginosa*

area	L*	a*	b*	c*
variegated	41.13 ± 2.70a	33.75 ± 3.59a	−8.42 ± 0.70a	34.78 ± 3.65a
non-variegated	50.73 ± 0.49b	26.07 ± 0.56a	−16.08 ± 0.26b	30.63 ± 0.61a

Different letters indicate significant differences in chromaticity values between areas at *P* < 0.05

L* indicates lightness; a* indicates the red-green axis; b* indicates the yellow-blue axis; and C* indicates chroma

Correlation analysis revealed significant relationships among the flower color parameters of *I. uliginosa* (*n* = 15), as presented in Table 2. The lightness value (L*) was significantly negatively correlated with both the yellow-blue parameter (b*) (r = −0.981, *P* < 0.01) and the red-green parameter (a*) (*r* = −0.757, *P* < 0.05). Furthermore, a* showed a highly significant positive correlation with chroma (C*) (*r* = 0.967, *P* < 0.01). These findings indicate that as L* decreases, indicating darker coloration, both redness and yellowness increase simultaneously, and that the red component is the primary determinant of chroma.

**Table 2:** Correlation analysis of floral CIELAB color values

	L*	a*	b*	c*
L*	1			
a*	−0.757*	1		
b*	−0.981**	0.870**	1	
c*	−0.566	0.967**	0.716*	1

^*^ *P* < 0.05; ** *P* < 0.01

### Cellular morphology of variegated and non-variegated areas in *I. uliginosa*

#### Petal pigment distribution in variegated and non-variegated areas

**Fig. 2:**
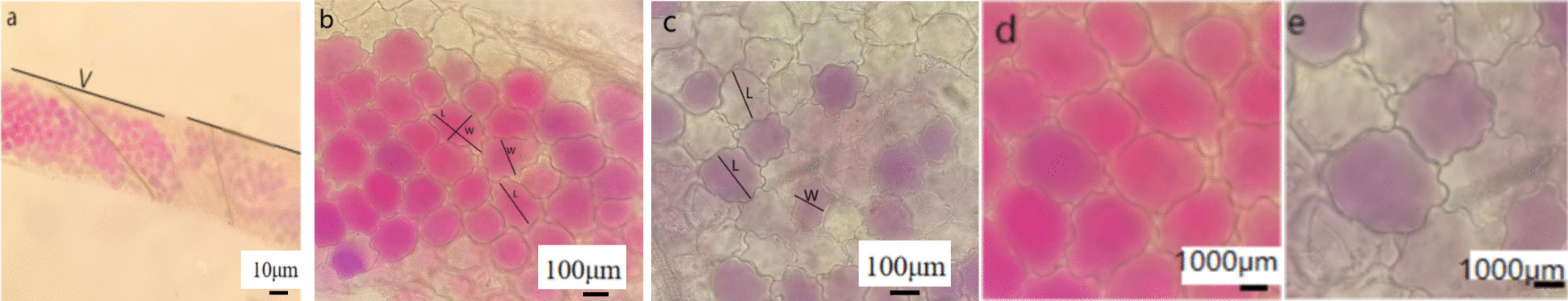
Microscopic observation of petal epidermal cells in *I. uliginosa*. **A** Transverse section of a variegated petal showing the variegated (V) and non-variegated (nV) regions. Scale bar = 10 μm. **B**, **C** Magnified view of upper epidermal cells from the variegated (**B**) and non-variegated (**C**) regions at 200 × magnification, showing pigment-cell distribution. L, length; W, width. Scale bars = 100 μm. **D**, **E** Comparison of cell morphology between the variegated (**D**) and non-variegated (**E**) areas. Scale bars = 1,000 μm

**Fig. 3:**
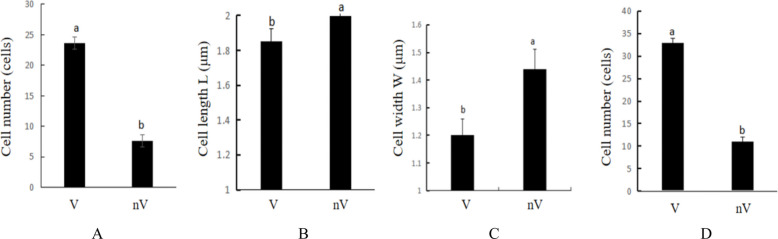
Quantitative analysis of epidermal cell characteristics in variegated (V) and non-variegated (nV) regions of *I. uliginosa* petals. **A** Number of pigment-containing cells per 200 × 200 μm field. **B** Cell length (L). **C** Cell width (W). **D** Total cell number per field

Cell numbers in the variegated and non-variegated areas were assessed using both freehand sections (Fig. 2) and paraffin sections. At 200 × magnification within a 200 × 200 μm field of view, marked differences in cell number were observed between the two tissue types. In the freehand sections (Fig. 3A), approximately 24 cells were observed in the variegated areas, compared with about 8 cells in the non-variegated areas. In the paraffin sections (Fig. 3D), the variegated areas contained roughly 33 cells, whereas the non-variegated areas contained approximately 11 cells. Taken together, the results from both methods consistently showed that the number of pigment cells in the variegated areas was approximately three times that in the non-variegated areas, with strong agreement between the two datasets. This spatial heterogeneity in cell-number distribution represents an important cytological basis underlying the visually distinct variegated phenotype.

Cell length (Fig. 2B) and width (Fig. 2C) were measured, and the results are shown in Figs. 3B and C. In the variegated areas, mean cell length was approximately 1.9 μm (corresponding to an actual length of about 17 μm), whereas in the non-variegated areas, mean cell length was approximately 2.0 μm (actual length about 18 μm). Mean cell width in the variegated areas was approximately 1.2 μm (actual width about 15 μm), compared with approximately 1.5 μm (actual width about 16 μm) in the non-variegated areas. Cells in the non-variegated areas were significantly longer than those in the variegated areas, suggesting that cells in the non-variegated regions may be at a more advanced developmental stage or may exhibit a distinct pattern of cell elongation. Although more numerous, cells in the variegated areas were shorter, which may be associated with active cell division and a lower degree of differentiation. Furthermore, cells in the non-variegated areas were significantly wider than those in the variegated areas, and the width difference (14%) was more pronounced than the length difference (8%). These findings indicate that cells in the non-variegated areas display a flattened and enlarged morphology, whereas those in the variegated areas appear short and compact.

Collectively, these findings suggest that pigment accumulation in the variegated areas (V) results from an increased number of pigment-accumulating cells rather than from cell expansion. Cells in the variegated regions are characterized by small size (short and narrow), high density, and elevated metabolic activity, making them well suited for rapid anthocyanin biosynthesis and accumulation. In contrast, cells in the non-variegated areas are larger (long and wide), less abundant, and presumably metabolically less active, suggesting that they primarily serve structural and protective roles with limited capacity for pigment production.

#### Petal epidermal cell morphology

**Fig. 4:**
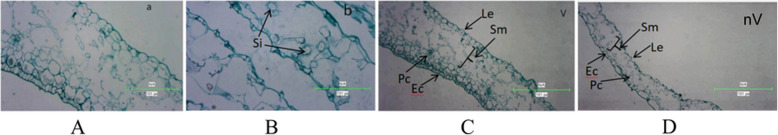
Histological characterization of pigment-cell distribution in the dorsal petal of *I. uliginosa. ***A** Cross-section of the variegated area showing the overall tissue structure. **B** Magnified view of the epidermis and subepidermis in the variegated area; arrows indicate stomata (Si). **C**, **D** Comparative cross-sections of the variegated (V) and non-variegated (nV) areas, respectively. Le, lower epidermis; Sm, spongy mesophyll; Ec, epidermal cell; Pc, pigment cell. Note the dense, plump pigment cells (Pc) in the variegated area compared with the sparse, flattened cells in the non-variegated area. All sections were stained with Safranin-Fast Green and observed under bright-field microscopy. Scale bars = 100 μm

As shown in Fig. 4, the upper epidermis and subepidermal layers of the variegated areas (V) contained abundant, plump, and nearly spherical pigment cells (Pc). These cells were densely arranged and exhibited high pigment content. In contrast, pigment cells in the non-variegated areas (nV) were sparse, flattened, and contained little pigment, representing the direct histological basis for the formation of purplish-red spots on the dorsal petals of *I. uliginosa*. Pigment accumulation occurred predominantly within the one to three cell layers immediately beneath the upper epidermis (Pc), consistent with an epidermal pigment-cell type of variegation mechanism. This pattern resembles that observed in ornamental flowers such as petunia and morning glory and is consistent with the vacuolar anthocyanin accumulation model. Cells in the variegated areas, including pigment cells (Pc), epidermal cells (Ec), and subepidermal cells (Sm), generally exhibited a plump, rounded morphology and dense arrangement, suggesting high metabolic activity, well-developed vacuoles, and efficient anthocyanin biosynthesis and storage capacity. Conversely, cells in the non-variegated areas appeared flattened and loosely arranged, indicative of lower metabolic activity. These observations suggest that during early floral development, specific epidermal precursor cells in certain regions may preferentially differentiate into pigment-accumulating cells, whereas cells in the non-variegated regions remain weakly pigmented or unpigmented.

The histological observations, namely, a threefold higher number of pigment cells in the variegated areas than in the non-variegated areas, were fully consistent with the previously measured color parameters (L* = 41.13 in the variegated areas vs. 50.73 in the non-variegated areas, *P* < 0.01; a* = 33.75 in the variegated areas vs. 26.07 in the non-variegated areas). This close agreement at the cellular level provides a mechanistic explanation for the dark purplish-red coloration of the variegated areas and the pale appearance of the non-variegated regions.

### Pigment content analysis of variegated *I. uliginosa*

#### Pigment content in variegated and non-variegated areas

**Fig. 5:**
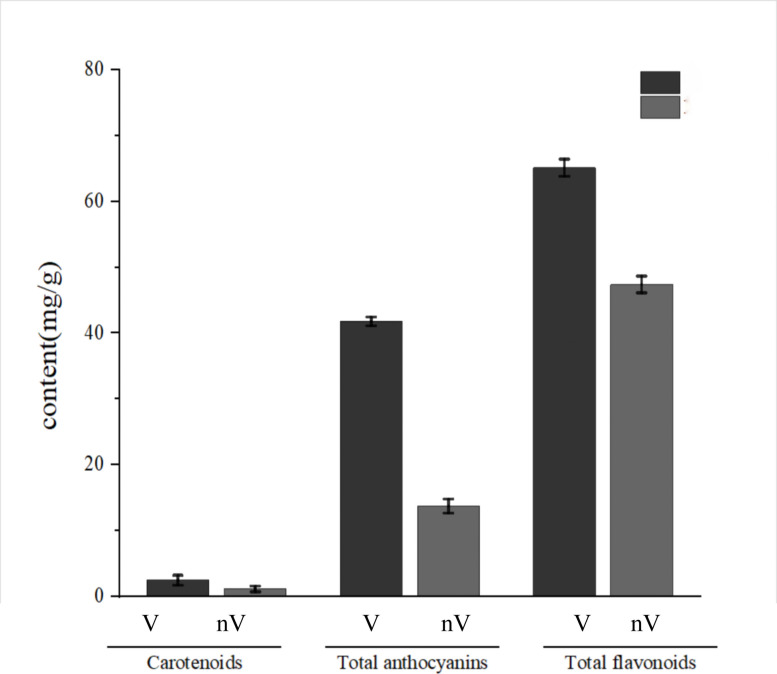
Comparison of pigment contents between variegated (V) and non-variegated (nV) regions of *I. uliginosa* petals. Contents of carotenoids, total anthocyanins, and total flavonoids were determined in V and nV regions. Data are presented as mean ± SD (*n* = 3). Asterisks indicate significant differences between V and nV regions (*P < 0.05, **P < 0.01)

As shown in Fig. 5, the anthocyanin content in variegated areas (41.30 mg/g) was approximately threefold higher than that in non-variegated areas (13.87 mg/g). As water-soluble pigments accumulated in vacuoles, anthocyanins are primarily responsible for the high-saturation purplish-red coloration observed in the variegated regions. Flavonoid content was also elevated in variegated areas (65.38 mg/g) compared to non-variegated areas (45.05 mg/g), representing an approximately 1.5-fold increase, although the magnitude of this difference was smaller than that observed for anthocyanins. As byproducts of the anthocyanin biosynthesis pathway and potential copigments, flavonoids may contribute to the stabilization of anthocyanin structures, enhance color stability (including resistance to light and heat), and provide ultraviolet protection. Carotenoid content, while substantially lower than that of anthocyanins and flavonoids, was approximately twofold higher in variegated areas (1.03 mg/g) than in non-variegated areas (0.51 mg/g). Despite their low abundance, carotenoids may fine-tune the final hue of the variegated pattern by superimposing red–orange tones, shifting the purplish-red coloration toward a warmer spectrum rather than a cool pure purple.

#### Semi-quantitative analysis of floral pigments in variegated *I. uliginosa*

**Fig. 6:**
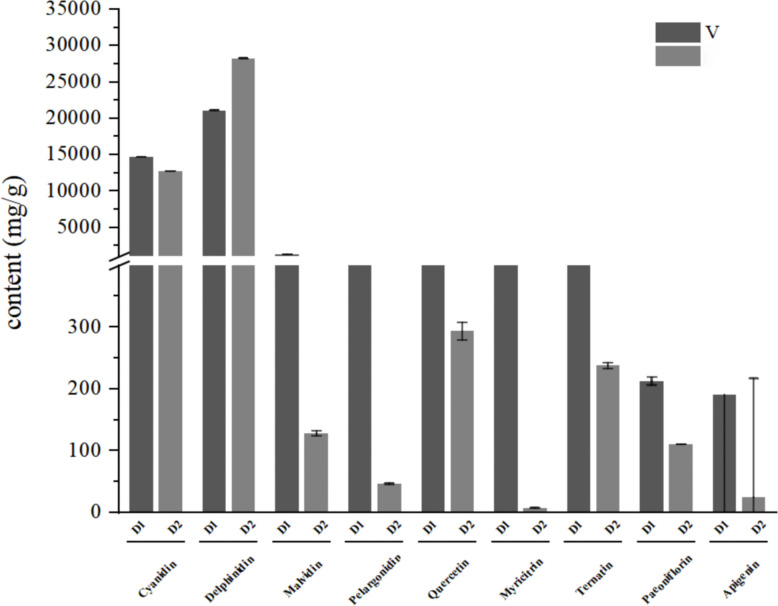
Relative contents of anthocyanidin and flavonoid aglycones in variegated (V) and non-variegated (nV) regions of *I. uliginosa* petals. (Content is 10.^4^)

As shown in Fig. 6, with the exception of quercetin, the contents of the remaining eight pigments were significantly higher in the variegated areas than in the non-variegated areas of *I. uliginosa*. Among these, the differences in delphinidin, pelargonidin, and peonidin were particularly pronounced. The elevated levels of these pigments in the variegated areas are likely major contributors to the darker and redder coloration observed, indicating that the pigmentation pattern results from the coordinated accumulation of multiple anthocyanin-related compounds rather than from the specific enrichment of a single pigment type. Delphinidin, pelargonidin, and peonidin therefore appear to be important contributors to coloration in the variegated areas. These findings reveal clear metabolic divergence between the variegated and non-variegated tissues: the variegated areas are characterized by stronger anthocyanin accumulation and deeper purplish-red coloration, whereas the non-variegated areas exhibit markedly lower anthocyanin content and a paler appearance, likely owing to the predominance of flavonols or other colorless or pale yellow compounds. Based on these observations, we suggest that variegated pattern formation in *I. uliginosa* is associated with activation of the anthocyanin biosynthetic pathway in the variegated regions, whereas this pathway is comparatively less active in the non-variegated areas.

### Transcriptomic analysis

#### RNA sequencing and de novo assembly

Transcriptome sequencing was conducted on the variegated (V) and non-variegated (nV) areas of *I. uliginosa* at full anthesis. A total of approximately 91.14 million raw reads were generated from the two samples, corresponding to about 13.76 GB of raw data. Following stringent quality filtering, approximately 90.31 million clean reads were retained, representing about 13.40 GB of clean data, with the proportion of effective reads exceeding 99% in each sample. The sequencing error rate was below 0.03% for all samples, Q30 values exceeded 92.64%, and GC content ranged from 44.68% to 44.78% (Table 3). These quality metrics indicate that the sequencing data were of high quality and suitable for subsequent transcriptome assembly and differential expression analysis.

**Table 3:** Efficiency of VIGS-mediated gene silencing in* I. uliginosa*

	**Number of injected clones**	**Surivival rate (%)**	**Konckdown rate (%)**
Positive control(EV)	30	68	
Treatment group	30	65	70

Thirty plants were injected for each treatment. The survival rate was similar between Positive Control (PC) and *IuMYB114*-silenced group (68% vs. 65%). Silencing efficiency was determined as the percentage of surviving plants showing faded petal color phenotype (70%)

The assembled sequences exhibited a broad length distribution, with marked variation in sequence abundance across different length intervals. Most sequences were concentrated in the shorter length ranges. Specifically, sequences in the 0–2,000 bp interval were the most abundant, indicating that short assembled sequences predominated in the dataset. As sequence length increased, the number of sequences in the corresponding intervals gradually decreased; however, considerable numbers of sequences were still retained in the 2,000–4,000 bp, 4,000–6,000 bp, and 6,000–8,000 bp intervals. Notably, some sequences were also detected in long-length intervals exceeding 10,000 bp (e.g., 10,000–12,000 bp, 14,000–16,000 bp, 16,000–18,000 bp, and 18,000–20,000 bp), suggesting that some transcripts may represent near-full-length structures or contain long untranslated regions (UTRs).

This overall distribution pattern is consistent with typical characteristics of de novo eukaryotic transcriptome assemblies, namely a predominance of short sequences accompanied by a proportion of medium- to long-length transcripts. These findings provide a foundation for subsequent transcript assembly evaluation, gene-structure annotation, and differential expression analysis (Figure S6).

#### Functional annotation

**Fig. 7:**
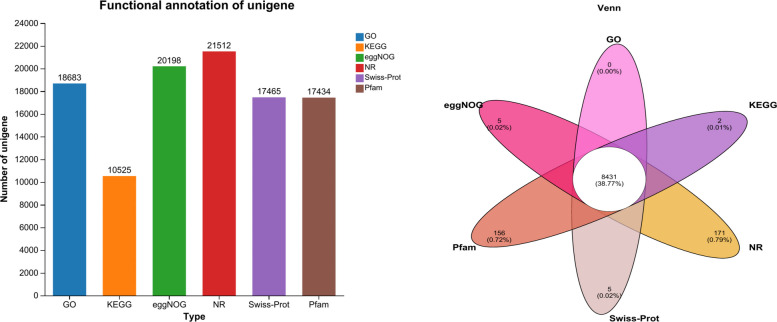
Functional annotation of unigenes in the *I. uliginosa* transcriptome. (Left) Number of unigenes annotated in public databases. (Right) Venn diagram showing the overlap of unigenes annotated across five databases (GO, KEGG, eggNOG, Swiss-Prot, and Pfam). The central number indicates unigenes annotated in all five databases

Statistical analysis was performed on all transcripts obtained from RNA sequencing, and their annotation status across multiple databases was compared, as illustrated in Fig. 7. The left panel shows the number of unigenes annotated in each of the six databases individually. The right panel presents a Venn diagram depicting the overlap of unigene annotations across the six databases, with a total of 8,431 unigenes commonly annotated in all six. The numbers of uniquely annotated unigenes in each database were as follows: GO (0), KEGG (2), NR (171), Swiss-Prot (5), Pfam (156), and eggNOG (5).

**Fig. 8:**
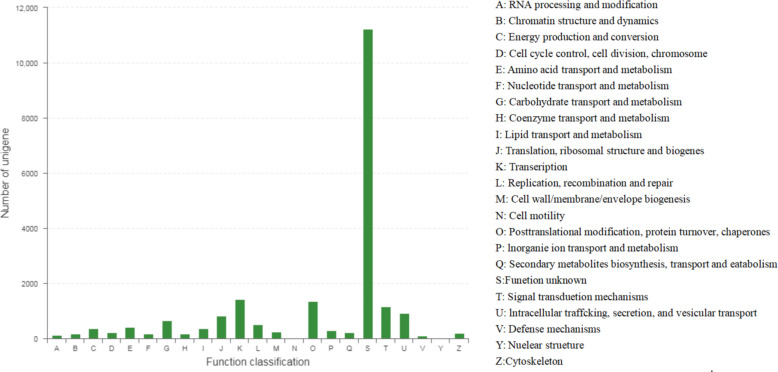
EggNOG functional classification of unigenes in the* I. uliginosa* transcriptome

The biological functions of *I. uliginosa* genes were systematically characterized through annotation against the COG, GO, and KEGG databases. COG annotation assigned functional categories to approximately 126,000 unigenes, representing 58.73% of annotated genes, which were grouped into four major functional modules. Within the information storage and processing module, genes involved in transcription (K category), replication, recombination and repair (L category), and translation (J category) were most abundant, with approximately 12,000, 10,000, and 8,000 unigenes, respectively. In the metabolism module, genes related to carbohydrate transport and metabolism (G category), amino acid transport and metabolism (E category), and secondary metabolite biosynthesis (Q category) predominated, with approximately 8,000, 8,000, and 4,000 unigenes, respectively. Within the cellular processes and signaling module, post-translational modification (O category) and signal transduction (T category) each comprised approximately 6,000 unigenes. Among other functional categories, cell wall/membrane/envelope biogenesis (M category) and inorganic ion transport (P category) contained approximately 4,000 and 3,500 unigenes, respectively. Notably, the bimodal distribution characterized by prominent peaks in transcription-related and replication/repair-related genes suggests the presence of a sophisticated transcriptional regulatory network and robust genome stability maintenance mechanisms in this species (Fig. 8).

**Fig. 9:**
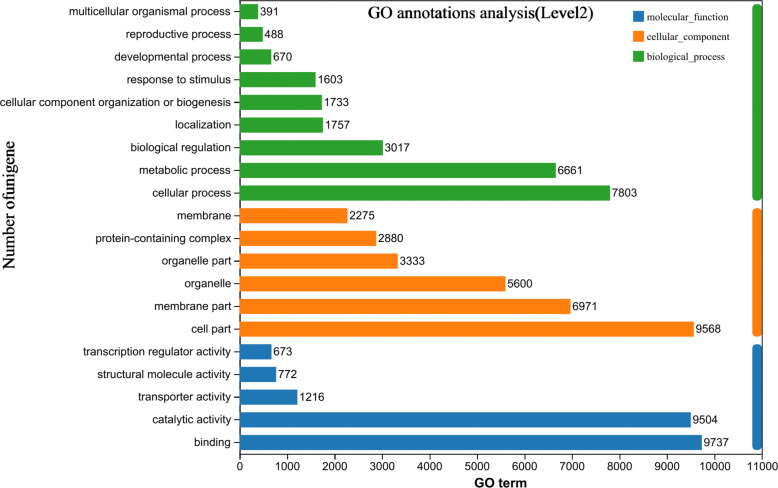
GO functional classification of unigenes in the *I. uliginosa* transcriptome at Level 2

GO annotation further revealed the distribution of 2,731 GO terms across the three ontologies. Within the biological process category, cellular process (7,803 unigenes) and metabolic process (6,611 unigenes) were the most highly represented terms. In the cellular component category, cell part (9,568 unigenes) and organelle (6,971 unigenes) were the most abundant terms. Within the molecular function category, binding (9,737 unigenes) and catalytic activity (9,504 unigenes) predominated, indicating extensive involvement of these genes in fundamental cellular activities and metabolic functions (Fig. 9).

KEGG annotation provided a comprehensive pathway-level perspective on biological functions. A total of 10,759 unigenes (27.23% of all annotated unigenes) were assigned to six major branches, including metabolism and genetic information processing, encompassing 20 secondary categories. Among these, carbohydrate metabolism (800 unigenes) and translation (1,143 unigenes) represented the most abundant subcategories within the metabolism and genetic information processing branches, respectively. Notably, 220 unigenes were classified under the biosynthesis of other secondary metabolites category, which includes genes involved in anthocyanin and flavonoid biosynthesis. This result highlights the active genetic basis of pigment synthesis and antioxidant metabolism in *I. uliginosa* (Figure S4).

#### Differential gene expression analysis

**Fig. 10:**
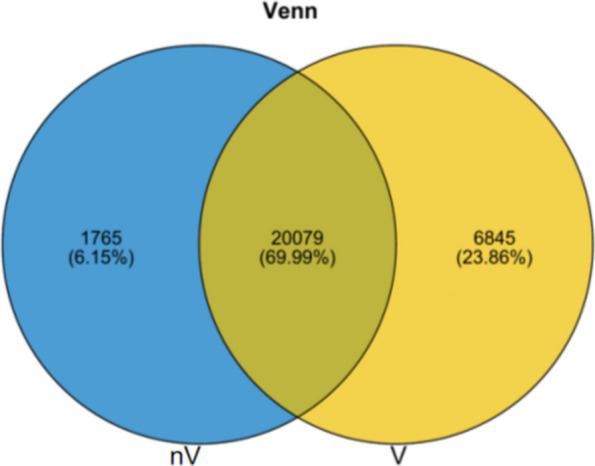
Venn diagram of differentially expressed genes (DEGs) between variegated (V) and non-variegated (nV) regions of *I. uliginosa* petals

Based on the distinct pigmentation patterns of the dorsal petals, *I. uliginosa* samples were categorized into two areas: variegated (V) and non-variegated (nV). Venn diagram analysis was employed to visualize the shared and unique genes/transcripts between the two sample types, thereby elucidating their transcriptional relationships. A total of 21,844 genes were detected in the nV sample, whereas 26,924 genes were detected in the V sample. As illustrated in Fig. 10, 20,079 genes were co-expressed in both samples. Additionally, 1,765 genes were specifically expressed in the nV sample, and 6,845 genes were specifically expressed in the V sample.

#### Enrichment analysis of differentially expressed genes

Differentially expressed genes (DEGs) between the variegated (V) and non-variegated (nV) areas of *I. uliginosa* dorsal petals were identified using the criteria |log2(fold change)|> 1 and adjusted P value < 0.005. Scatter plots were generated to visualize gene expression profiles, with each point representing the expression level of a single gene under the same experimental conditions. In these plots, red points indicate significantly upregulated genes, blue points indicate significantly downregulated genes, and black points denote genes without significant differential expression. Genes with lower expression levels tend to cluster near the origin, whereas those that deviate more strongly from the diagonal line between the two samples exhibit more pronounced expression differences.

**Fig. 11:**
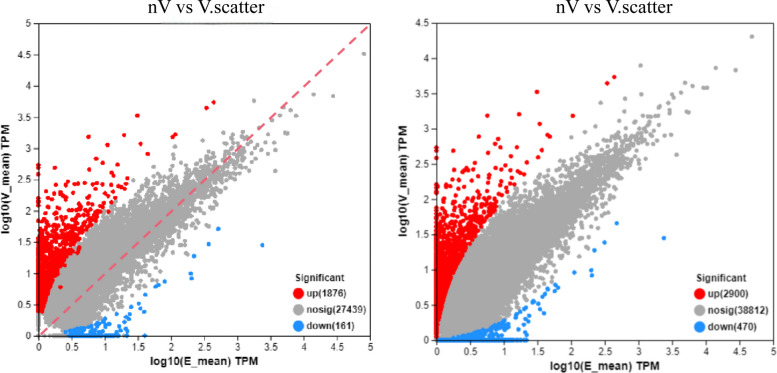
Unigene and Transcript Scatter plot of expression difference

As shown in Fig. 11, the unigene-based scatter plot revealed 1,876 upregulated genes and 161 downregulated genes in the V vs. nV comparison. In the right panel, the transcript-based scatter plot identified 2,900 upregulated genes and 470 downregulated genes. For subsequent differential expression analysis, the unigene dataset was selected as the reference for further investigation.

#### Identification of transcription factors

**Fig. 12:**
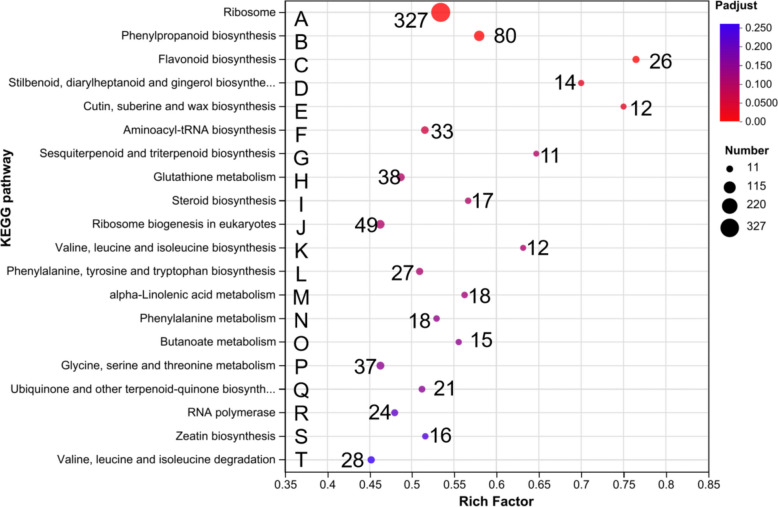
Family statistics of transcription factors

As shown in Fig. 12, a total of 20 transcription factor families were identified among the differentially expressed genes between the variegated and non-variegated dorsal petal regions of *I. uliginosa*. Among these transcription factor families, MYB, AP2/ERF, bHLH, WRKY, NAC, and MADS-box members showed relatively high abundance, suggesting that these transcription factors may participate in the regulatory processes associated with the formation of variegated and non-variegated dorsal petal regions.

**Fig. 13:**
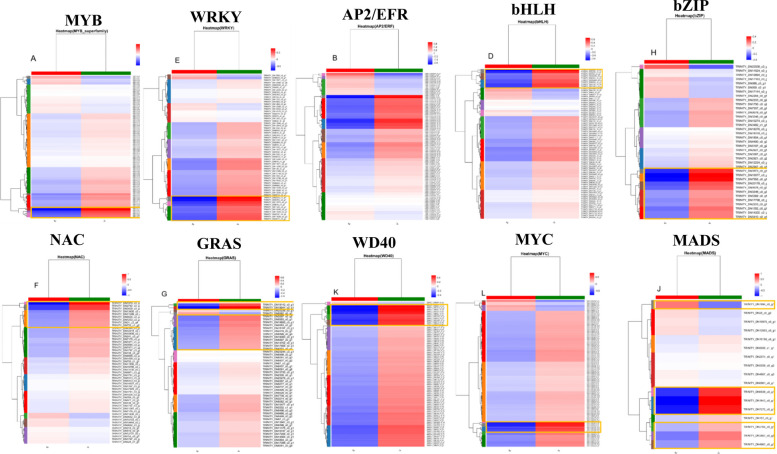
Expression profiles of differentially expressed genes (DEGs) in ten transcription factor families between variegated (V) and non-variegated (nV) regions of *I. uliginosa* petals

Hierarchical clustering analysis of transcription factor expression profiles in the variegated (V) and non-variegated (nV) areas of *I. uliginosa* is shown in Fig. 13. The clustering heatmap highlights genes with elevated expression levels in each sample type, facilitating the preliminary identification of candidate genes and the construction of gene sets for downstream analyses. Among the MYB transcription factor family, 10 genes exhibited pronounced expression differences between the V and nV samples. In the AP2/ERF family, 25 genes showed substantial expression divergence, while the bHLH, WRKY, NAC, GRAS, bZIP, MADS, and WD40 families contained 9, 10, 9, 14, 12, 6, and 11 genes with marked expression differences, respectively.

#### Candidate genes associated with anthocyanin biosynthesis

**Fig. 14:**
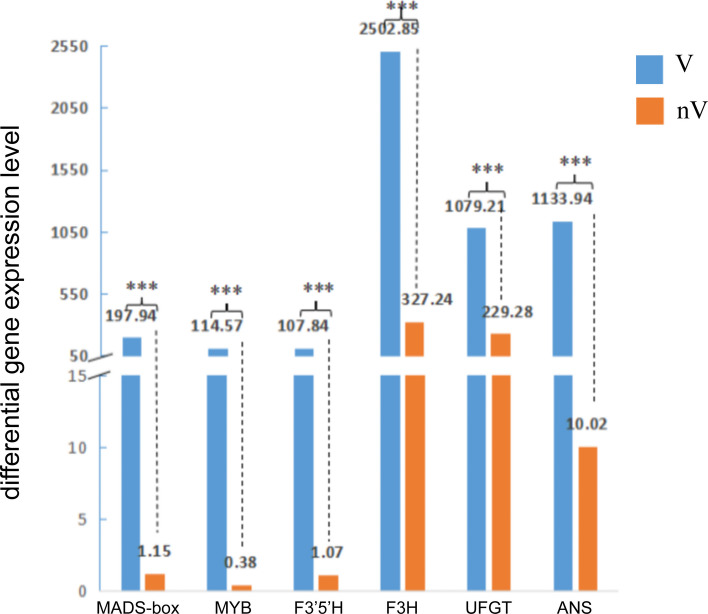
Validation of differentially expressed genes (DEGs) involved in anthocyanin biosynthesis and transcriptional regulation by qRT-PCR

Analysis of the transcriptome data revealed the expression profiles of genes involved in anthocyanin biosynthesis in *I. uliginosa*. A total of six candidate genes showing significant differential expression between the variegated (V) and non-variegated (nV) areas were identified, including members of the MYB, MADS-box, *F3H*, and *F3′5'H* families. Specifically, two MYB genes, two MADS-box genes, one *F3H* gene, and one *F3′5'H* gene displayed significantly higher transcript levels in the variegated areas than in the non-variegated areas (Fig. 14). These differentially expressed genes are likely to play important roles in regulating the variegated pigmentation pattern observed in *I. uliginosa* petals. Among these candidate genes, Fig. 14 specifically presents genes directly associated with anthocyanin accumulation in the variegated and non-variegated dorsal petal regions and selected for qRT-PCR validation.

#### Validation of candidate gene expression by qRT-PCR

**Fig. 15:**
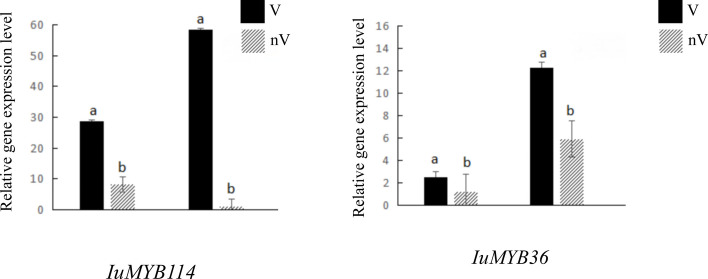
Relative expression of *IuMYB114*, *IuMYB36* genes in different floral organs of *I.uliginosa*

Given the critical role of MYB transcription factors in regulating anthocyanin biosynthesis, two MYB family genes, *IuMYB114* and *IuMYB3*6, were selected for validation by quantitative real-time PCR (qRT-PCR). Expression analysis showed that the transcript levels of both genes were generally consistent between the RNA-seq and qRT-PCR datasets, confirming the reliability of the sequencing results. Comparative analysis across different flowering stages (initial, middle, and full flowering stages) and petal regions (variegated vs. non-variegated areas) demonstrated that both genes were consistently more highly expressed in the variegated areas than in the non-variegated areas at all stages examined. As shown in Fig. 15, *IuMYB114* expression was markedly higher than that of *IuMYB36*, particularly at the middle flowering stage, at which its expression in the variegated areas was 53.1-fold higher than in the non-variegated areas. Based on the well-established regulatory function of MYB family members in flower pigmentation and the pronounced expression advantage of *IuMYB114*, this gene was selected as the key candidate for subsequent functional validation aimed at elucidating the molecular mechanism underlying variegated petal formation in *I. uliginosa*.

#### Functional validation of *IuMYB114* in variegated pattern formation in *I. uliginosa*

**Fig. 16:**
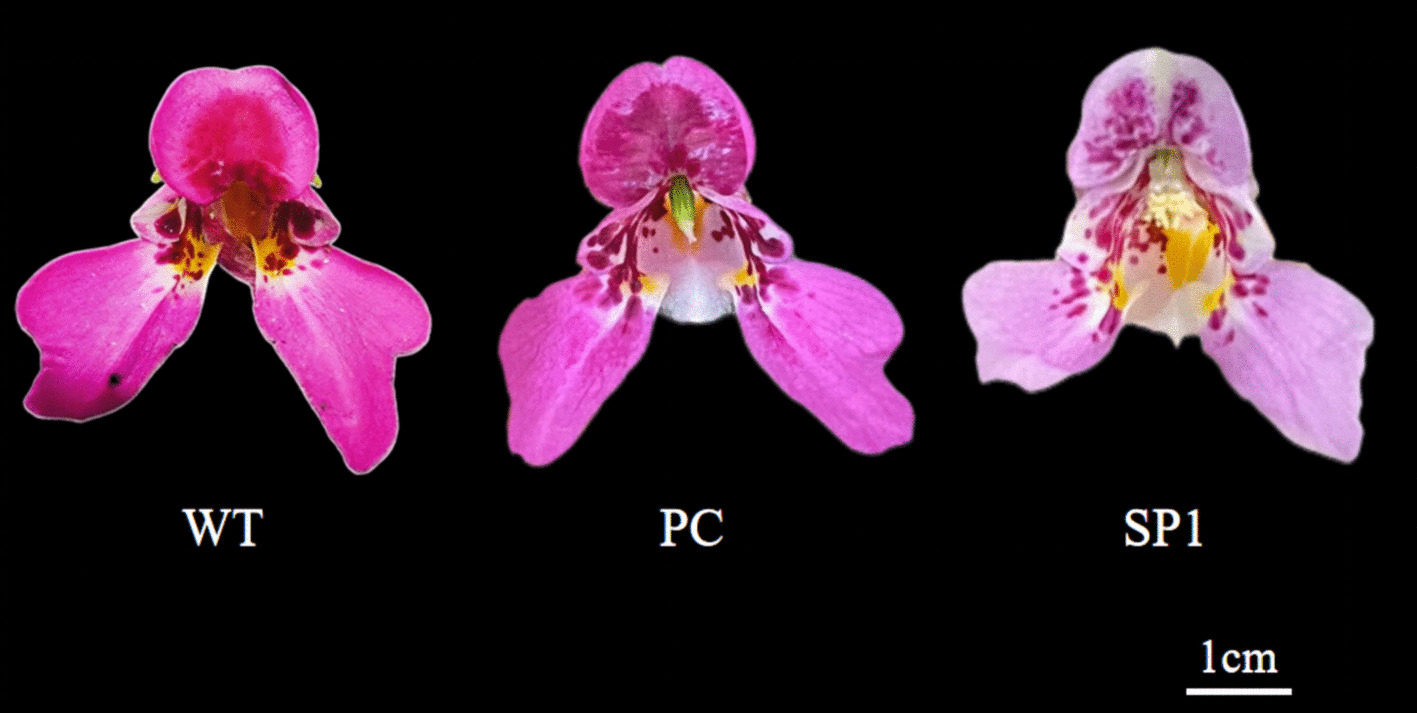
Phenotypic analysis of *IuMYB114*-silenced *I. uliginosa* flowers. Flowers of wild type (WT), Positive Control (PC), and *IuMYB114*-silenced line (SP1) are shown. Silencing of *IuMYB114* resulted in faded petal color and reduced anthocyanin accumulation in SP1 compared to WT and PC. Bar = 1 cm

To investigate the molecular mechanism underlying variegated pattern formation in *I. uliginosa*, the candidate transcription factor gene *IuMYB114* was subjected to functional validation. Phenotypic changes in variegated plants were first observed approximately two weeks after Agrobacterium-mediated infiltration. As shown in Fig. 16, control plants infiltrated with the empty vector displayed no obvious phenotypic differences from wild-type plants. In contrast, *IuMYB114*-silenced plants (SP1) exhibited a marked reduction in the size of the variegated areas, a decrease in overall flower size, and a shift in flower color from deep pink to light pink.

To further assess gene-silencing efficiency, phenotypic changes were statistically analyzed in the infiltrated plants. The survival rates were 68% for the empty-vector control group and 65% for the treatment group, and the target-gene silencing efficiency was 70% (Table 3). These results demonstrate that the TRV-based VIGS system was successfully established in variegated *I. uliginosa* and achieved effective silencing of *IuMYB114*.

**Fig. 17:**
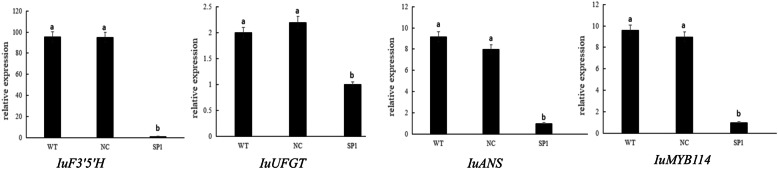
Relative expression of *IuMYB114* and anthocyanin structural genes in different floral organs of variegated *I. uliginosa*

To investigate the impact of *IuMYB114* silencing on the anthocyanin biosynthetic pathway, three structural genes, *IuF3′5'H*, *IuANS*, and *IuUFGT*, that showed significant differential expression in the transcriptome data were selected, and their expression levels in different floral organs were examined by qRT-PCR. The results showed that in *IuMYB114*-silenced plants, *IuMYB114* expression was downregulated by approximately 8- to ninefold. Concurrently, the expression levels of *IuANS*, *IuUFGT*, and *IuF3′5'H* also decreased to varying degrees, with *IuF3′5'H* showing the most pronounced reduction (Fig. 17). After silencing of *IuMYB114*, the pigmentation in the variegated dorsal petal regions became significantly lighter compared with that of the control plants. In addition, the expression levels of several anthocyanin biosynthetic genes were significantly decreased after *IuMYB114* silencing. These results suggest that *IuMYB114* may participate in the transcriptional regulation of anthocyanin biosynthesis in the variegated and non-variegated dorsal petal regions of *I. uliginosa*.

## Discussion

Floral variegation is a complex petal trait whose underlying mechanisms involve epidermal-cell microstructure, pigment distribution, and physiological and biochemical processes. Previous studies have shown that pigment accumulation in variegated petal areas is often associated with specific epidermal cell layers. For instance, in *Phalaenopsis aphrodite*, pigments accumulate predominantly in the upper epidermis of the variegated regions [46]. In *Paeonia rockii*, the number and morphology of pigment cells also play important roles in determining flower color [47]. In the present study, observations from both freehand and paraffin sections revealed that pigments in both the variegated (V) and non-variegated (nV) areas of *I. uliginosa* petals were primarily localized in the upper epidermis. Notably, the number of pigment cells in the variegated areas was significantly higher, approximately threefold, than that in the non-variegated areas. Cells in the variegated regions exhibited a plump, rounded, and densely arranged morphology, whereas those in the non-variegated regions were larger, irregularly shaped, and more loosely arranged. These findings suggest that differences in the number, size, shape, and packing density of pigment cells constitute an important structural basis for floral variegation.

Spatial differences in pigment composition and content form the biochemical basis of flower-color variegation. In this study, anthocyanin, flavonoid, and carotenoid contents in the variegated areas of *I. uliginosa* were significantly higher than those in the non-variegated areas, and pigment accumulation was positively correlated with color intensity. This pattern is consistent with pigment-enrichment patterns reported in *Lilium brownii* [48] and *Raphanus sativus* [49]. The marked differences in anthocyanin and flavonoid accumulation between the variegated and non-variegated regions suggest that these two classes of compounds are key metabolites driving color differentiation. Anthocyanins, as the primary chromogenic components of the flavonoid pathway, are widely recognized as critical determinants of flower-color diversity and variegated pattern formation [50]. In *Nigella orientalis* and *Phalaenopsis aphrodite*, the formation of variegated patterns depends on the high-level accumulation of anthocyanins specifically within the variegated areas [46–51].

The coloration conferred by anthocyanins is largely determined by their aglycone types. The most common anthocyanidin aglycones include cyanidin, pelargonidin, delphinidin, peonidin, petunidin, and malvidin [52]. Among these, pelargonidin and cyanidin are associated with red pigmentation, whereas peonidin is associated with purplish-red hues. Delphinidin, petunidin, and malvidin confer blue to blue-violet tones [53–55]. Previous studies have shown that specific variegated patterns often result from the combined accumulation of multiple aglycones. For example, the purple-red patch at the base of the passionflower corolla is rich in both delphinidin and cyanidin [56]. Consistent with this general pattern, the present study showed that several anthocyanin-related compounds, especially delphinidin-, pelargonidin-, and peonidin-related compounds, were more abundant in variegated than in non-variegated areas. Their enrichment in the variegated regions likely contributes to the darker and redder appearance of these tissues, indicating that coloration in the variegated areas arises from the coordinated accumulation of multiple pigments rather than from a single dominant compound. Furthermore, four pigment compounds, cyanidin-3-galactose-galactoside, dihydroquercetin, trimethoxyflavone, and chrysophanol-8-O-glucoside, were found to accumulate specifically in the variegated areas. These compounds may contribute to pigmentation and associated metabolic processes in the variegated regions.

Differences in gene expression within the anthocyanin and flavonoid biosynthesis pathways are an important molecular basis for flower-color variation. In *Orychophragmus violaceus* and *Rhododendron rex*, differentially expressed genes were significantly enriched in the anthocyanin and flavonoid biosynthesis pathways [57, 58]. The functional enrichment results obtained in the present study are consistent with these findings, indicating that the variegated phenotype of *I. uliginosa* also arises from marked alterations in gene expression within these pathways. Although sampling at earlier developmental stages may provide more direct information for identifying the initial regulatory genes involved in the formation of variegated and non-variegated dorsal petal regions, precise sampling during early floral development in *I. uliginosa* is technically challenging because the spatial boundaries are not yet clearly established and the tissue volume is extremely limited. Therefore, samples at full anthesis were selected in this study to ensure accurate tissue separation and sampling reliability.

Transcriptome analysis identified a total of 20 transcription factor families, including MYB, AP2/ERF, and bHLH, among which the MYB family exhibited the most pronounced expression differences. MYB transcription factors, as key regulators of anthocyanin biosynthesis, have been functionally characterized in numerous plant species. The first MYB gene shown to regulate anthocyanins, *ZmC1*, was identified in *Zea mays* [27]. Subsequent studies, from the regulation of variegated patterns by *PsMYB12* in *Paeonia suffruticosa* [59] to the induction of red flower coloration by MYB factors in *Rhododendron simsii* [60], have collectively underscored the pivotal role of MYB transcription factors in anthocyanin biosynthesis and patterning across diverse plant lineages.

In this study, qRT-PCR validation revealed that the expression pattern of *IuMYB114* was especially prominent. The MYB114 subfamily, which is known to function as a positive regulator of anthocyanin biosynthesis, has been extensively characterized in Arabidopsis thaliana, pear, and apple [25, 61, 62]. To further assess its function, we silenced *IuMYB114* using virus-induced gene silencing (VIGS) and observed a marked reduction in the size of the variegated areas in *I. uliginosa* petals, accompanied by a shift in flower color from deep pink to light pink. These phenotypic changes indicate that loss of *IuMYB114* activity strongly affects pigmentation in the variegated regions. After silencing, the expression of key downstream structural genes in the anthocyanin biosynthesis pathway, namely *IuANS*, *IuUFGT*, and *IuF3′5'H*, was downregulated to varying degrees, with IuF3′5'H showing the most pronounced reduction. This result is consistent with previous findings in alfalfa, where downregulation of *F3′5'H* affected flower-color formation [63], and supports the interpretation that *IuMYB114* contributes to anthocyanin biosynthesis and spatial pigment distribution by positively regulating the expression of *IuF3′5'H*, *IuANS*, and *IuUFGT*. Collectively, these results indicate that *IuMYB114* is an important positive regulator associated with both pigment intensity and the extent of the variegated areas in *I. uliginosa* flowers. However, the underlying regulatory network is likely more complex, and the precise molecular mechanisms governing variegated pattern formation warrant further investigation. In the present study, the TRV-based VIGS system was established based on our previous work in *I. uliginosa*, in which the empty TRV vector showed no obvious effects on plant growth, floral morphology, or developmental processes. Therefore, the same validated VIGS system was adopted in this study using the empty pTRV2 vector as the control. Although inclusion of unrelated-gene controls would further strengthen experimental rigor by excluding potential non-specific silencing effects, the current study primarily focused on preliminary candidate gene screening, and more comprehensive control strategies will be incorporated in future mechanistic studies.

The silencing results, combined with expression analysis, provide preliminary evidence supporting the involvement of *IuMYB114* in anthocyanin regulation. However, the current data are insufficient to conclusively demonstrate direct transcriptional regulation of downstream structural genes by *IuMYB114*. Direct molecular interactions, including promoter binding activity and transcriptional activation, remain to be experimentally verified through yeast one-hybrid assays, electrophoretic mobility shift assays (EMSA), dual-luciferase reporter assays, and other molecular approaches. Therefore, the regulatory relationship identified in this study should currently be considered as a potential rather than definitive transcriptional interaction.

Several limitations of this study should be acknowledged. First, the transcriptomic comparison was restricted to variegated and non-variegated dorsal petal regions at a single developmental stage, and therefore does not resolve temporal regulatory dynamics. Second, only one candidate regulator, *IuMYB114*, was functionally validated; additional MYB, bHLH, and structural-gene candidates, as well as direct promoter interactions, remain to be examined. Third, although the pigment analyses clearly distinguished the two tissue types, more comprehensive targeted metabolite profiling would further refine the contributions of individual anthocyanin derivatives. Accordingly, the regulatory model proposed here should be regarded as a supported working model that will benefit from further molecular and biochemical validation.

Overall, the present study provides preliminary evidence supporting the involvement of *IuMYB114* and anthocyanin biosynthetic genes in the formation of variegated and non-variegated dorsal petal regions in *Impatiens uliginosa*.

The pigment analysis performed in this study mainly aimed to preliminarily identify the major pigment pathways associated with the formation of variegated and non-variegated dorsal petal regions. The observed consistency between pigment accumulation patterns and transcriptomic enrichment of anthocyanin biosynthesis genes provides strong support for the involvement of anthocyanin metabolism in regional pigmentation. However, semi-quantitative pigment analysis alone is insufficient to directly link *IuUFGT* expression with specific anthocyanin glycosides. More comprehensive targeted metabolomic analyses, such as LC–MS/MS-based profiling, will be performed in subsequent mechanistic studies. Importantly, the current work primarily focuses on candidate gene identification and initial functional screening rather than comprehensive mechanistic validation. Therefore, incorporation of unrelated-gene controls in VIGS experiments, direct molecular validation of transcriptional regulation, and targeted metabolomic analyses will be systematically addressed in our subsequent studies.

## Conclusion

In this study, integrated cytological, physiological, pigment, transcriptomic, and VIGS analyses revealed significant differences between the variegated and non-variegated dorsal petal regions of *I. uliginosa*, indicating that differential anthocyanin accumulation is closely associated with floral variegation formation. Several structural genes involved in anthocyanin biosynthesis, including *CHS*, *CHI*, *F3H*, *DFR*, *ANS*, and *UFGT*, as well as multiple transcription factors, were identified as potential candidates associated with regional pigmentation. Among them, *IuMYB114* showed a strong correlation with anthocyanin accumulation, and VIGS analysis provided preliminary evidence suggesting that *IuMYB114* may participate in the transcriptional regulation of anthocyanin biosynthesis in the variegated and non-variegated dorsal petal regions. These findings provide an important foundation for further elucidating the molecular mechanisms underlying floral variegation formation in *Impatiens* species.

## Supplementary Information

Supplementary Material 1.

Supplementary Material 2.

Supplementary Material 3.

## Data Availability

The datasets generated during the current study are available in the NCBI Sequence Read Archive (SRA) repository under BioProject accession number PRJNA1404166, https://www.ncbi.nlm.nih.gov/bioproject/PRJNA1404166.

## Abbreviations

CIELAB: Commission Internationale de l’Eclairage L*a*b* color space
DEG: Differentially expressed gene
DP: Dorsal petal
FAA: Formalin-acetic acid-alcohol
FDR: False discovery rate
FPKM: Fragments per kilobase of transcript per million mapped reads
GO: Gene Ontology
KEGG: Kyoto Encyclopedia of Genes and Genomes
LS: Lower sepal
nV: Non-variegated area
OD600: Optical density at 600 nm
PC: Positive control
qRT-PCR: Quantitative real-time PCR
RSEM: RNA-Seq by Expectation–Maximization
SRA: Sequence Read Archive
TPM: Transcripts per million
TRV: Tobacco rattle virus
UTR: Untranslated region
V: Variegated area
VIGS: Virus-induced gene silencing
WP: Wing petal
