# Supplementary material for: Transcriptional profiling of anthocyanin biosynthesis in variegated and non-variegated dorsal petal regions of Impatiens uliginosa
Source: BMC Plant Biol. 2026 Jun 3;26:1321. doi: 10.1186/s12870-026-09116-2 (PMC13449508; doi:10.1186/s12870-026-09116-2)
Supplement: Supplementary file 1 [file 12870_2026_9116_MOESM1_ESM.docx]

**T1: Key to the Pigments of Floral Variegation in** *Impatiens uliginosa*

D1: Variegated area of upper petal; D2: Non-variegated area of upper petal

| No. | Name | Formula | **Identification Result** |
| --- | --- | --- | --- |
| 1 | Lawsone ( 2-Hydroxy-1,4-naphthoquinone) | C_10_H_6_O_3_ | √ |
| 2 | （2-Methoxy-1,4-naphthoquinone ） Methoxy-lawsone | C_11_H_8_O_3_ | √ |
| 3 | 1,4-Naphthoquinone | C_10_H_6_O_2_ | × |
| 4 | Dantron**（**1,8-Dihydroxyanthraquinone**）** | C_16_H_12_O_5_ | × |
| 5 | Balsaminone A | C_21_H_22_O_5_ | √ |
| 6 | Balsaminone B | C_27_H_22_O_10_ | × |
| 7 | Balsaminone C | C_21_H_12_O_5_ |  |
| 8 | Impatienolate | C_22_H_12_O_6_Na_2_ | add Na √ |
| 9 | Impatienol | C_22_H_14_O_6_ | add Na √ |
| 10 | 2-Methoxy-3-(2-hydroxyethyl)-1,4-naphthoquinone | C_13_H_12_O_4_ | add Na √ |
| 11 | Cyanidin | C_15_H_11_O_6_ | √ |
| 12 | Cyanidin-3-O-glucoside | C_21_H_21_O_11_ | √ |
| 13 | Cyanidin 3-acetylgalactosylgalactoside | C_30_H_37_O_16_ | × |
| 14 | Cyanidin 3-galloylgalactoside | C_28_H_25_O_15_ | × |
| 15 | Cyanidin 3-digalactoside | C_27_H_29_O_16_ | D1√ |
| 16 | Cyanidin 3-trigalactoside | C_33_H_39_O_21_ | × |
| 17 | Cyanidin 3-sambubioside | C_26_H_29_O_15_ | × |
| 18 | Cyanidin 3-rhamnoside / Cyanidin 3-rutinoside | C_27_H_31_O_15_ | × |
| 19 | Cyanidin methyl ether | C_17_H_14_O_7_ | √ |
| 20 | Delphinidin | C_15_H_11_O_7_ | √ |
| 21 | Delphinidin 3-glucoside | C_21_H_21_O_12_ | √ |
| 22 | Delphinidin 3-rhamnoside | C_21_H_21_O_11_ | √ |
| 23 | Delphinidin 3-arabinoside | C_20_H_19_O_11_ | D1× |
| 24 | Delphinidin 3,5-diglucoside | C_27_H_31_O_17_ | × |
| 25 | Delphinidin 3-rhamnosylgalactoside | C_28_H_33_O_15_ | D2× |
| 26 | Delphinidin 3-galactoside-5-acetylgalactoside | C_30_H_35_O_17_ | × |
| 27 | Delphinidin 3-acetylglucoside | C_24_H_25_O_12_ | √ |
| 28 | Malvidin | C_17_H_15_O_7_ | √ |
| 29 | Malvidin 3-arabinoside | C_22_H_23_O_11_ | √ |
| 30 | Malvidin 3-galactoside | C_23_H_25_O_12_ | √ |
| 31 | Malvidin 3,5-diglucoside | C_29_H_35_O_17_ | × |
| 32 | Malvidin 3-rhamnoside-5-glucoside | C_29_H_35_O_16_ | √ |
| 33 | Pelargonidin([M+H]⁺ peak matches mass of C₁₅H₁₀O₄ + H) | C_15_H_11_O_4_ | × |
| 34 | Pelargonidin | C_15_H_11_O_5_ | D2√ |
| 35 | Leucopelargonidin | C_15_H_14_O_6_ | √ |
| 36 | Pelargonidin 3,5-diglucoside | C_27_H_31_O_15_ | D2× |
| 37 | Pelargonidin 3-glucoside | C_21_H_21_O_10_ | √ |
| 38 | Petunidin | C_16_H_13_O_7_ | √ |
| 39 | Petunidin 3,5-diglucoside | C_28_H_33_O_17_ | × |
| 40 | Peonidin | C_16_H_13_O_6_ | √ |
| 41 | Peonidin 3-glucoside / Peonidin 3-galactoside | C_22_H_23_O_11_ | √ |
| 42 | Peonidin 3,5-diglucoside / Petunidin 3-rhamnoside-5-glucoside | C_28_H_33_O_16_ | × |
| 43 | Delphinidin 3''-O－（6”-O-acety-β－galactopyranoside） | C_23_H_23_O_13_ | √ |
| 44 | Delphinidin 3-O-rhamnosyl-5-O-galactoside | C_27_H_41_O_16_ | × |
| 45 | Delphinidin 3''-O－（2”－O－galloyl－6”-O-acety-β－galactopyranoside） | C_30_H_27_O_17_ | × |
| 46 | Cyanidin 3-O-acetylrutinoside | C_29_H_32_O_16_ | √ |
| 47 | Flavone | C_15_H_10_O_2_ | D2√ |
| 48 | 7-Hrdroxy-neoflavone | C_15_H_10_O_3_ | D2× |
| 49 | Apigenin | C_15_H_10_O_5_ | D1√ |
| 50 | Luteolin/Kaempferol/Scutellarein | C_15_H_10_O_6_ | √ |
| 51 | Quercetin | C_15_H_10_O_7_ | √ |
| 52 | Flavanone | C_15_H_12_O_2_ | √ |
| 53 | Pinocembrin | C_15_H_12_O_4_ | add Na √ |
| 54 | Naringenin/Pinobanksin | C_15_H_12_O_5_ | × |
| 55 | Dihydrokaempferol /Sakuranetin/Eriodictyol/Aromadendrin | C_15_H_12_O_6_ | D2√ |
| 56 | Dihydroquercetin | C_15_H_12_O_7_ | D1√ |
| 57 | Catechin | C_15_H_14_O_6_ | √ |
| 58 | Gallocatechin | C_15_H_14_O_7_ | √ |
| 59 | Leucodelphinidin | C_15_H_14_O_8_ | √ |
| 60 | Pseudobatigenin | C_16_H_10_O_5_ | × |
| 61 | Tectochrysin | C_16_H_12_O_4_ | √ |
| 62 | Kaempferol 3-monomethyl ether/Isokaemferide | C_16_H_12_O_6_ | √ |
| 63 | Quercetin 3-monomethyl ether | C_16_H_12_O_7_ | √ |
| 64 | Alpinetin/7-Methyl liquiritigenin/Strobopinin/Cryptostrobin | C_16_H_14_O_4_ | add Na √ |
| 65 | Sakuranetin/Dihydrooroxylin/Angophorol | C_16_H_14_O_5_ | √ |
| 66 | Hesperidin/Hesperetin/Homoeriodictyol | C_16_H_14_O_6_ | √ |
| 67 | (-)-Epicatechin-3'-O-methylether〔(-)-EC-3'-O-Me〕 | C_16_H_16_O_6_ | D2× |
| 68 | Karanjonol | C_17_H_10_O_4_ | D1× |
| 69 | Maximaisoflavone A | C_17_H_10_O_6_ | × |
| 70 | Irisone A | C_17_H_12_O_6_ | add Na D1× |
| 71 | 6-Methoxy-2-p-tolyl-chromen-4-one | C_17_H_14_O_3_ | √ |
| 72 | Pterocapin | C_17_H_14_O_5_ | D1√ |
| 73 | Kampferol-3,4'-dimethyl ether/Dehydrodihymocarpin A | C_17_H_14_O_6_ | √ |
| 74 | 5,7-Dihydroxyflavone (Chrysin) | C_17_H_14_O_7_ | √ |
| 75 | Axillarin | C_17_H_14_O_8_ | add Na √ |
| 76 | 5,7,3’,4’-Tetrahydroxy-6,8-dimethoxyflavonol | C_17_H_14_O_9_ | √ |
| 77 | Liquiritigenin dimethyl ether/comptonin | C_17_H_16_O_4_ | D1× |
| 78 | Didymocarpin-A | C_17_H_16_O_6_ | √ |
| 79 | Dalbergiphenol | C_17_H_18_O_3_ | √ |
| 80 | R-latifolin | C_17_H_18_O_4_ | D2√ |
| 81 | 7,2‘-Dihydroxy-3’,4’-dimethoxyisoflavan | C_17_H_18_O_5_ | √ |
| 82 | Dihydrokaranjin mehyl ether | C_18_H_14_O_4_ | √ |
| 83 | Maximaisoflavone D | C_18_H_14_O_6_ | add Na √ |
| 84 | Maximaisoflavone F | C_18_H_14_O_7_ | add Na √ |
| 85 | 5,7,4‘-Trimethoxyflavone | C_18_H_16_O_5_ | D1√ |
| 86 | Eupatilin/Pachypodol | C_18_H_16_O_7_ | D1√ |
| 87 | 5,7-Dihydroxy-4’-methoxy-6,8-dimethyl-anthocyanidin | C_18_H_17_O_4_ | √ |
| 88 | Capensinidin | C_18_H_17_O_7_ | D1√ |
| 89 | Dolineone | C_19_H_12_O_6_ | D2√ |
| 90 | Isokanugin | C_19_H_16_O_7_ | add Na √ |
| 91 | Melisimplin | C_19_H_16_O_8_ | add Na √ |
| 92 | Methylophiopogonanone | C_19_H_20_O_5_ | √ |
| 93 | Kanakugin | C_19_H_20_O_6_ | √ |
| 94 | Licoisoflavone B | C_20_H_16_O_6_ | × |
| 95 | Elliptone | C_20_H_16_O_8_ | add Na D2× |
| 96 | Neorautenol | C_20_H_18_O_4_ | √ |
| 97 | Glepidotin | C_20_H_18_O_5_ | √ |
| 98 | Licoisoflavone A | C_20_H_18_O_6_ | √ |
| 99 | Irisflorentin | C_20_H_18_O_8_ | add Na √ |
| 100 | Kaempferol 4‘-O-xyloside | C_20_H_18_O_10_ | × |
| 101 | Quercetin 3-O-xyloside | C_20_H_18_O_11_ | D2√ |
| 102 | Myricetin 3-O-xyloside | C_20_H_18_O_12_ | × |
| 103 | Glabranin/Isobavachin/Glabranin | C_20_H_20_O_4_ | √ |
| 104 | Sophoraflavanone | C_20_H_20_O_5_ | √ |
| 105 | Euchrestraflavanone | C_20_H_20_O_6_ | √ |
| 106 | Isosinensetin | C_20_H_20_O_7_ | √ |
| 107 | Artemetin | C_20_H_20_O_8_ | √ |
| 108 | 5’-Hydroxy-6,7,2’,4’,5’-pentamethoxyflavonol/Naringenin 4‘-O-β-D-xylopyranoside | C_20_H_20_O_9_ | √ |
| 109 | Isoflavone A | C_21_H_18_O_4_ | √ |
| 110 | Apigenin 7-O-glucuronide | C_21_H_18_O_11_ | × |
| 111 | Quercetin 3-O-β-D-glucuronide | C_21_H_18_O_13_ | × |
| 112 | Edulenane | C_21_H_20_O_4_ | √ |
| 113 | Purpurin | C_21_H_20_O_5_ | √ |
| 114 | Cavenegrin A-1 | C_21_H_20_O_6_ | √ |
| 115 | Suphoronol | C_21_H_20_O_7_ | √ |
| 116 | Chrysophanol 8-O-glucoside | C_21_H_20_O_9_ | D1√ |
| 117 | Neoliquiritin | C_21_H_22_O_9_ | D1√ |
| 118 | Naringenin-7-O-glucoside | C_21_H_22_O_10_ | D2√ |
| 119 | Myricitrin (Myricetin 3-rhamnoside) | C_21_H_20_O_12_ | √ |
| 120 | [Nobiletin](http://www.so.com/link?m=aVETcA6rfl9HuiEN/7rZXDpQEORmJJa7CWHboddxNut1cfkOPYl0MhT1ZjPxfxuegc25gEozPYYzcT0JT+KvrGUHKbHRC7Jq1sB3vWhDgmhDkpqN4) | C_21_H_22_O_8_ | √ |
| 121 | Kaempferol 3-rhamnoside | C_21_H_20_O_10_ | √ |
| 122 | Kaempferol 4’-O-glucoside | C_21_H_20_O_11_ | √ |
| 123 | Quercetin 3-O-rhamnoside | C_21_H_20_O_11_ | √ |
| 124 | Quercetin 3-O-glucoside | C_21_H_20_O_12_ | √ |
| 125 | Naringenin 5-O-glucoside | C_21_H_22_O_10_ | D2√ |
| 126 | Myricetin 3-O-glucoside | C_21_H_20_O_13_ | add Na D1√ |
| 127 | 7,3’,4’-Trihydroxy-5-O-a-L-rhamnopyranosylsoflavone | C_21_H_20_O_10_ | √ |
| 128 | Isochamanetin/Chamanetin | C_22_H_18_O_5_ | √ |
| 129 | Dimethoxylalpinum isoflavone | C_22_H_20_O_5_ | √ |
| 130 | 7,3’,4’-Trihydroxy-5-O-b-D-(2”-acetyl)-xylopyranosyl-isoflavone | C_22_H_20_O_11_ | add Na × |
| 131 | Rhynchospermin | C_22_H_22_O_7_ | √ |
| 132 | Tectoridin | C_22_H_22_O_11_ | √ |
| 133 | Isorhamnetin 3-O-glucoside | C_22_H_22_O_12_ | √ |
| 134 | Rotenone | C_23_H_22_O_6_ | × |
| 135 | Myricetin3-O-(6"-O-ACETYL)- β-D-GALACTOPYRANOSIDE | C_23_H_22_O_14_ | √ |
| 136 | Myricetin3-O-(2"-O-ACETYL)-rhamnopyranoside | C_23_H_22_O_13_ | √ |
| 137 | Kaempferol 3-O-(3"-O-acetyl)-alpHa-L-rhamnoside | C_23_H_22_O_11_ | √ |
| 138 | Malaccol | C_23_H_24_O_7_ | √ |
| 139 | Ternatin | C_23_H_24_O_12_ | √ |
| 140 | Dihrdrorotenonic acid | C_23_H_26_O_6_ | D1× |
| 141 | Paeoniflorin | C_23_H_28_O_11_ | √ |
| 142 | Iridin | C_24_H_26_O_13_ | add Na × |
| 143 | Hydnocarpin | C_25_H_20_O_9_ | √ |
| 144 | Millettin | C_25_H_22_O_6_ | D2√ |
| 145 | Silychristin | C_25_H_22_O_10_ | add Na D2× |
| 146 | Angustone B | C_25_H_24_O_6_ | D1√ |
| 147 | Sanggenon A | C_25_H_24_O_7_ | D2√ |
| 148 | Mundulin | C_25_H_26_O_4_ | √ |
| 149 | Lupinifolin/Minimiflorin | C_25_H_26_O_5_ | √ |
| 150 | Ototinin | C_25_H_26_O_6_ | D2× |
| 151 | Kushenol C | C_25_H_26_O_7_ | D2× |
| 152 | Apigenin 6-C-β-D-xylosyl-8-C-β-D-glucoside | C_25_H_26_O_13_ | √ |
| 153 | Linderatone | C_25_H_28_O_4_ | √ |
| 154 | Bonannione | C_25_H_28_O_5_ | √ |
| 155 | Kuwanon E/Nymphaeol/Kuwanon F | C_25_H_28_O_6_ | √ |
| 156 | Kushenol | C_25_H_28_O_8_ | √ |
| 157 | Picroside III | C_25_H_30_O_13_ | × |
| 158 | Methoxy-hydrocarpin | C_26_H_22_O_10_ | × |
| 159 | Broussoflavonol A | C_26_H_26_O_7_ | √ |
| 160 | Apiin | C_26_H_28_O_14_ | add Na D2√ |
| 161 | Sophoraisoflavanone B | C_26_H_30_O_5_ | √ |
| 162 | (2R)naringenin-5-O-βD—xylopyranosyl(1—6)β-D- glucop)rranoside | C_26_H_30_O_14_ | × |

T2：**Sample Assembly**

| **Type** | **Unigene** | **Transcript** |
| --- | --- | --- |
| Total number | 29476 | 42182 |
| Total base | 32162082 | 50646588 |
| Largest length (bp) | 7726 | 7726 |
| Smallest length (bp) | 201 | 201 |
| Average length (bp) | 1091.13 | 1200.67 |
| N50 length (bp) | 1683 | 1736 |
| E90N50 length (bp) | 1999 | 1897 |
| Fragment mapped percent(%) | 77.91 | 88.91 |
| GC percent (%) | 41.29 | 41.08 |
| TransRate score | 0.41 | 0.47 |
| BUSCO score | C:62.3%[S:58.1%;D:4.2%] | C:62.3%[S:58.1%;D:4.2%] |

Table 3 Summary of sequencing data of *I. uliginosa* transcriptome

| Sample | Raw reads | Raw bases | Clean reads | Clean bases | Error rate(%) | Q20(%) | Q30(%) | GC content(%) |
| --- | --- | --- | --- | --- | --- | --- | --- | --- |
| E | 45519450 | 6873436950 | 45151284 | 6700084534 | 0.0255 | 97.88 | 93.58 | 44.68 |
| V | 45621578 | 6888858278 | 45159352 | 6700494648 | 0.0265 | 97.46 | 92.64 | 44.78 |

S1: Unigene Annotation Statistics Chart

S2: NR Annotation Statistics Chart

S3: Identification of transcription factor families

S4: Pathway categorization statistics histogram

S5: 3D chromaticity plot of variegated and non-variegated regions in *I. uliginosa*

S6: Length distribution of unigenes
